# Managing Recurrent Postictal Worsening of Psychosis in Clozapine‐Treated Schizophrenia: A Case Report

**DOI:** 10.1155/crps/8712049

**Published:** 2026-02-23

**Authors:** Rafae Alam, Robert O. Cotes, David R. Goldsmith

**Affiliations:** ^1^ Emory University School of Medicine, Atlanta, Georgia, USA, emory.edu; ^2^ Department of Psychiatry and Behavioral Sciences, Emory University School of Medicine, Atlanta, Georgia, USA, emory.edu

## Abstract

Postictal worsening of psychosis can be described as the development or exacerbation of psychotic symptoms following a seizure. Patients on clozapine for treatment‐resistant schizophrenia are at an increased risk for seizures and subsequent postictal worsening of psychosis. Because patients with schizophrenia may experience ongoing psychotic symptoms, it may be difficult to appreciate the development of postictal worsening of psychosis in these patients. We present the case of a 49‐year‐old woman with a longstanding history of schizophrenia treated with clozapine who presented to the emergency department (ED) after experiencing a seizure. In the following days, she and her mother reported an exacerbation of psychotic symptoms, which was ultimately attributed to a postictal worsening of psychosis. She was successfully treated after being prescribed an increased nightly dose of clozapine. This dose was eventually reduced to the usual dose she had been taking prior to the postictal exacerbation of psychosis. We also briefly describe a recurrent episode the patient experienced approximately 1 year later. This case contributes to the literature by offering one potential management strategy for postictal worsening of psychosis in a patient with a primary psychotic disorder.

## 1. Introduction

The atypical antipsychotic drug clozapine has been shown to be more efficacious than other antipsychotics in the treatment of schizophrenia [1]. The use of clozapine has been limited by a number of side effects, most notably its risk of severe neutropenia, which occurs in around 1% of patients [2]. Seizures are another potential side effect that occurs in ~1%–5% of patients on clozapine, with the risk being dose‐dependent and influenced by clozapine plasma levels [3]. Seizures of any etiology can induce or exacerbate psychosis [4]. Postictal psychosis is a specific phenomenon that can occur in patients with epilepsy in the week following a seizure or a cluster thereof. It most commonly occurs within 72 h after the last seizure and tends to present with more manic symptoms compared to other presentations of psychosis [5]. Although there is a paucity of empirical evidence on the topic, expert opinion suggests that episodes of postictal psychosis can be prevented or shortened by the introduction of antipsychotics at the first signs of an impending episode [6]. Moreover, regarding the management of postictal psychosis in individuals with a primary seizure disorder, optimal seizure control is the primary target of therapeutic intervention [7]. Managing psychotic and epileptic symptoms in the same patient involves a bidirectional risk. Many antipsychotics can lower the seizure threshold. Uncontrolled studies in patients without epilepsy suggest a dose‐dependent seizure risk of ~0.1%–1.5%, with clozapine associated with the highest risk. At the same time, several antiseizure medications (ASMs) have been associated with a small but measurable risk of psychotic symptoms, with reported prevalences generally in the 1%–2% range: topiramate 0.8%, vigabatrin 2.5%, zonisamide 1.9%–2.3%, levetiracetam 0.3%–0.7%, and gabapentin 0.5% [8]. While a number of studies and case reports have characterized the presentation and management of postictal psychosis in epileptic patients [9], there have been none to our knowledge in patients with a primary psychotic disorder in addition to a possible secondary diagnosis of epilepsy. Here, we describe a patient with a history of schizophrenia who experienced a seizure while on clozapine, followed by a worsening of baseline psychotic symptoms in the subsequent days. The patient was later found by her neurologist to likely have focal‐onset epilepsy. This case provides an example of how postictal exacerbation of psychosis in someone with a primary psychotic disorder can be treated while highlighting the need for further research into the optimal management of this condition. Moreover, the unique contributions and considerations of a patient on clozapine with postictal worsening of psychosis are discussed.

## 2. Case Presentation

The patient was a 49‐year‐old woman with schizophrenia treated with 200 mg of clozapine nightly who presented to the emergency department (ED) in the morning after experiencing a seizure. The episode began with her face twitching and her head turning, followed by generalized tonic‐clonic movements that lasted for a few minutes. She was sitting in a chair during the episode and did not fall nor hit her head. No tongue trauma or incontinence was noted.

The patient began taking clozapine at age 17 years and experienced her first seizure a year later. The patient and her mother attributed that seizure to high clozapine levels although the level was unknown at the time. The patient did not experience another seizure until she was 43 years old, when her aunt had recently passed away and she was going through a period of significant stress. Then, at age 46 years, the patient experienced a third seizure when she was admitted to a rural hospital with an acute kidney injury. At the time of the present seizure, she had been prescribed 200 mg of lamotrigine twice daily for mood stabilization and seizure prophylaxis. Her parents reported that she vomited the lamotrigine on the morning of the seizure but was generally adherent to her medication regimen, which consisted of lamotrigine 400 mg/day and clozapine 200 mg/day, as well as mirabegron 50 mg/day and solifenacin 10 mg/day for urinary incontinence. She did not report starting any new medications or herbal supplements and never smoked tobacco or drank alcohol. Pertinent stressors included the recent passing of her dog, as well as her parents’ health problems, which they noted had troubled the patient.

During the mental status exam, the patient was oriented to self and to being in the hospital. However, she was slow to answer questions and was sometimes tangential in her answers. Her affect was blunted. The patient’s vital signs were unremarkable. Laboratory investigations including a comprehensive metabolic panel, complete blood count with differential, venous blood gas, serum ammonia, serum lactate, pregnancy testing, serum acetaminophen and salicylate levels, and urinalysis were all within reference values. Unfortunately, clozapine plasma concentration was not obtained, neither was any electroencephalography (EEG) performed in the ED, so the cause of the seizure remained unclear. Later in the day, the patient’s condition improved, and she became more alert. However, while her parents were at her bedside, she said that she did not feel safe going home, although she could not articulate why. The patient also complained of a headache, for which she was given 650 mg of acetaminophen. She was also given lamotrigine to take home. After further discussion with the patient and her family, the patient stated that she would be comfortable returning home if her deceased dog’s belongings were removed from the house. She was discharged with a referral to a follow‐up visit with her psychiatrist. Clozapine was continued at 200 mg nightly.

Before the psychiatric follow‐up visit 6 days later, the patient’s mother reported that the patient had been experiencing worsening psychosis and difficulty sleeping. At the follow‐up visit, her parents noted that the patient had been experiencing significant auditory hallucinations and paranoia at night that were preventing her from falling asleep despite adhering to her prescribed medication regimen. These symptoms were not present prior to the seizure episode. The patient shared that she had been continuing to feel stressed. She endorsed the nighttime hallucinations and paranoia that her parents reported, as well as similar symptoms during the day. She also noted that she sometimes felt as if there were lasers coming out of her eyes.

During the mental status examination, the patient was fully oriented to person, place, and situation but not to time. Attention, concentration, and memory were grossly intact but not formally tested. She appeared restless during the interview and was often moving around the room and switching chairs. She appeared irritable, and her thought process was not fully logical nor linear, but she was able to engage in conversation and denied suicidal or homicidal ideation. The patient’s vital signs were significant for mild hypertension (142/79 mmHg) and tachycardia (112/min). Blood samples drawn at the visit revealed a clozapine plasma level of 255 ng/mL and a lamotrigine plasma level of 6.4 mcg/mL (reference interval for epilepsy 2.5–15.0 mcg/mL).

The patient’s presentation was most consistent with a postictal increase of baseline psychosis. Postictal delirium was also considered. However, aside from brief disorientation to time, her attention appeared grossly intact, and no significant fluctuation in consciousness was noted, making delirium less likely. The decision was made to increase the clozapine dose from 200 to 250 mg nightly while keeping in close touch with the patient and her family.

The patient and her mother presented for another follow‐up visit 2 weeks later, where the patient reported that she was no longer experiencing paranoia, auditory hallucinations, or other psychotic symptoms. The patient also reported resolution of insomnia. Overall, the patient and her mother felt that the increased dose of clozapine had been very helpful. The patient was kept on the increased dose of 250 mg of clozapine nightly, but a plan to taper the medication back down to a dose of 200 mg nightly in the future was discussed. Over the next 3 months, the patient continued to report an absence of psychotic symptoms, and the clozapine dose was eventually reduced back to 200 mg nightly. The patient tolerated the decrease without any recurrence of psychotic symptoms or mood fluctuations.

Of note, approximately 1 year after the clinical course described above, the patient was admitted to the neurological intensive care unit for status epilepticus. A clozapine plasma level was not obtained during her admission, but her lamotrigine plasma level was 3.6 mcg/mL. While in the hospital, the patient was given her usual dose of 200 mg of lamotrigine twice daily and was additionally prescribed 100 mg of lacosamide twice daily. A continuous EEG revealed abnormal generalized spike/polyspike‐and‐wave discharges at 1–3 Hz, often with a predominance in the right frontal region, indicative of an increased risk of seizure. The EEG also revealed areas of right cerebral dysfunction that were assessed to likely be of structural and/or functional etiology. The patient underwent magnetic resonance imaging (MRI) of the brain, which showed no acute abnormality but did reveal chronic small‐vessel ischemic changes and diffuse cerebral atrophy. When the patient was seen by psychiatry 8 days after the seizure, she was provisionally diagnosed with postictal psychosis, and the clozapine dosage that she was prescribed was initially increased to 225 mg nightly, followed by another increase to 250 mg nightly 3 days later. At this dose, her clozapine level was 251 mcg/L. However, 6 days after the dose was increased to 250 mg nightly, she was noted to have periorbital myoclonus, raising the concern that the patient may have a clozapine‐induced seizure. The dose was decreased to 225 mg nightly, and she was discharged from the hospital the next day with a follow‐up appointment with her outpatient psychiatrist.

At the follow‐up appointment 3 weeks later, the clozapine dose was decreased to the typical dose that the patient had been taking, that is, 200 mg nightly, and her overall functioning and experience with psychosis ultimately returned to baseline. Of note, since the hospitalization for status epilepticus, the patient was followed by neurology in close collaboration with her primary psychiatrist. Per the assessment of her neurologist, the patient likely had focal‐onset epilepsy given her rare seizures that occurred typically but not exclusively at high clozapine levels. At her first neurology visit following the most recent hospitalization described, lamotrigine was increased to 250 mg twice daily, and a plan was made to taper off the lacosamide.

## 3. Discussion

The management of postictal worsening of psychosis in a patient with a primary psychotic disorder presents therapeutic challenges. In both episodes presented here, the seizures that the patient experienced were potentially secondary to clozapine, which had effectively supported her recovery while living with psychosis for over 30 years. Therefore, when the patient began experiencing a worsening of psychosis in the days following the seizures, a decision had to be made about decreasing, increasing, or maintaining the nightly clozapine dosage that she was prescribed. Although there was a risk that increasing the nightly dose of clozapine could induce a seizure, the decision was made to slightly increase the dose because of the distress caused by the worsening psychotic symptoms. Fortunately, in both episodes, the increased clozapine dose was associated with a reduction in the new psychotic symptoms that the patient was experiencing, though it is difficult to ascertain whether this was a direct consequence of the increased clozapine dose or simply the natural course of the acute psychotic symptoms. A major factor guiding the management of these recurrent episodes of postictal worsening of psychosis was the favorable response of the psychotic symptoms that the patient had previously experienced to clozapine, when other antipsychotics had failed. Although this patient’s experience suggests that clozapine may be particularly effective in managing psychotic symptoms in someone with postictal worsening of psychosis, there remains a paucity of literature to guide evidence‐based decision‐making in such cases.

An important consideration regarding a patient on clozapine for the treatment of a primary psychotic disorder who presents with increased psychotic symptoms following a seizure is whether the change represents an exacerbation of the primary psychotic disorder or a postictal phenomenon. Table 1 outlines several considerations related to a patient’s clozapine level that may assist in making this determination. Unfortunately, in both episodes of the case described here, clozapine levels were not obtained at the time of the initial presentation. Psychological stress may have also influenced our patient’s clozapine levels; however, its effect has not been directly examined in prior studies. Due to the limited evidence on this topic, it should be emphasized that this table serves only as one tool to assist physicians in managing complex patients with a primary psychotic disorder who have experienced a seizure [10–12].

**Table 1:** Key considerations differentiating primary psychotic exacerbation from postictal worsening of psychosis in a patient on clozapine.

Clinical consideration	Exacerbation of primary psychosis	Postictal psychosis
Clozapine levels	May be subtherapeutic	May be unchanged
Drug interactions	Medications added that reduce clozapine levels (e.g., carbamazepine and rifampin) [10]	Medications added that increase clozapine levels (e.g., fluvoxamine and ciprofloxacin) [10] or lower the seizure threshold (e.g., bupropion and clomipramine) [11]
Clozapine dose changes before seizure onset	Dose reduction preceding seizures	Dose increase preceding seizures
Changes in smoking habits	Increase in smoking intensity or return to smoking	Decrease in smoking intensity or smoking cessation
Physiological stress (e.g., infection) [12]	Return to baseline physiological stress after resolution of acute inflammation (due to risk of subtherapeutic clozapine levels if dose was reduced)	Increased physiological stress (if clozapine dose is not reduced during period of acute inflammation)

Although clinicians may avoid using clozapine in patients with epilepsy or a history of seizures, clozapine remains the gold standard for people with schizophrenia who have failed treatment with at least two other antipsychotics taken consistently, at an adequate dose, for an adequate duration [13]. A history of seizures is not an absolute contraindication for using clozapine but better understanding of the relationship between clozapine and seizures can aid in more effective management [14]. Clozapine is associated with a higher risk of seizures compared to other antipsychotics, with the risk of seizures during treatment estimated to be ~1%–5%. Seizure risk is dependent on the dose and plasma level, with some authors and guidelines suggesting that it becomes significant enough to warrant primary prophylaxis at doses above 600 mg/day or plasma levels above 1300 µg/L [15]. The Maudsley Guidelines note that seizures may occur at any time during treatment with clozapine. In the case of a seizure, they recommend stopping the drug for 1 day before restarting it with an ASM [16]. As acknowledged in these guidelines and illustrated in this case, there may be patients who experience such improvement in psychotic symptoms on clozapine that they, in collaboration with their prescriber, decide that it is appropriate to continue clozapine despite the risk of seizures. Nevertheless, in such cases, it is important that patients with a history of seizures are also treated with an appropriate ASM regimen. In some cases, collaboration with colleagues in neurology may be beneficial. Sodium valproate has shown good tolerability and some benefit in augmenting the efficacy of clozapine and could be an option in managing both treatment‐resistant schizophrenia and seizure risk. However, the increased risk of myocarditis and neutropenia with concurrent clozapine and sodium valproate use merit careful consideration, particularly during clozapine titration [17, 18]. Topiramate has also shown efficacy in this regard though its tolerability appears to be a concern, as evidenced by the relatively high all‐cause discontinuation rates reported in studies evaluating topiramate as an augmenting agent to clozapine [13]. Lamotrigine, which present patient had been prescribed for mood stabilization and seizure prophylaxis, has been found to provide clinically meaningful benefit to a subset of patients with persistent psychotic symptoms on clozapine [19]. However, a recent meta‐analysis found that lamotrigine had a small effect size and was not significantly better than a placebo in augmenting the effects of clozapine [20]. Of note, levetiracetam has been associated with a particularly high rate of psychiatric side effects and may not be the most prudent option for individuals experiencing psychosis [21].

The evaluation and management of postictal psychosis may benefit from a multidisciplinary team, or at least a framework incorporating both neurological and psychiatric perspectives. Patients experiencing psychotic symptoms in the context of a seizure can experience hallucinatory states that may be clinically indistinguishable from those experienced by patients with primary psychotic disorders. This can lead one to speculate on whether epilepsy and schizophrenia might share some underlying pathophysiology. Moreover, patients with focal epilepsy may undergo network‐level brain changes associated with the development of psychosis [22]. The patient presented here was started on clozapine for treatment of schizophrenia at a young age and before having experienced any seizures. Nevertheless, she had experienced multiple seizures since she was diagnosed with schizophrenia and was eventually thought to have focal‐onset epilepsy. It is possible that these seizures have had an enduring effect on the manifestation of the primary psychotic symptoms that she experienced. Although some patients may experience improvement in psychotic symptoms after a seizure, the patient presented here experienced increased psychotic symptoms. It is unclear why our patient experienced a worsening of her symptoms, though it is intriguing that postictal psychosis has been shown to more likely occur in people with epilepsy who also have an increased genetic predisposition to schizophrenia [5, 23].

Ultimately, this case underscores the need for further research into the management of patients with primary psychotic disorders who experience seizures and resulting postictal worsening of psychotic symptoms. Likewise, further research should seek to uncover the interplay between seizures and psychotic symptoms in both patients with epilepsy and primary psychotic disorders. Close collaboration between neurologists and psychiatrists is essential for this aim.

## Funding

No funding was received for this manuscript.

## Consent

Verbal informed consent was obtained from the patient for publication of this case report.

## Conflicts of Interest

Robert Cotes has received research funding (to institution) from Otsuka, Karuna, Boehringer Ingelheim, and Alkermes. He is a consultant to IQVIA, Boehringer Ingelheim, and Syneos Health on behalf of the Clozapine Product Manufacturers Group. He is a speaker and consultant to Saladax Biomedical. Rafae Alam and David Goldsmith have nothing to disclose.

## Data Availability

The data that support the findings of this study are available upon request from the corresponding author. The data are not publicly available due to privacy or ethical restrictions.
